# Psychometric Properties of the Depression, Anxiety, Stress Scales-21 (DASS-21) in a Portuguese Sample during the early stage of the COVID-19 pandemic

**DOI:** 10.1192/j.eurpsy.2024.333

**Published:** 2024-08-27

**Authors:** C. Laranjeira, M. A. Dixe, A. I. Querido

**Affiliations:** ^1^ School of Health Sciences; ^2^ciTechCare, Polytechnic University of Leiria, Leiria, Portugal

## Abstract

**Introduction:**

The COVID-19 global crisis has resulted in significant disruptions in the lives of students in higher education, leading to negative consequences for their academic achievements and general psychological well-being.

**Objectives:**

In this study, we sought to examine the psychometric properties of the Depression Anxiety Stress Scale-21 (DASS-21) among students in Portuguese higher education institutions during the initial phase of the COVID-19 pandemic and its efficacy in capturing mental health symptoms due to a global health crisis.

**Methods:**

In this cross-sectional study, a convenience sampling method was used to enlist a total of 1522 participants. The sample consisted of 75.1% women and 79.2% undergraduate students. Participants completed an electronic survey that was designed using the Depression Anxiety Stress Scale-21 (DASS-21) — a self-report instrument measuring anxiety, depression, and stress.

**Results:**

The findings of the study indicated a significant occurrence of depressive symptoms [≥10] (N = 434, 28.5%), anxiety symptoms [≥7] (N = 551, 36.2%), and stress symptoms [≥11] (N = 544, 35.7%). Based on the collected data, a Confirmatory Factor Analysis (CFA) was conducted in order to examine the factor structure of the scale. The analysis revealed a three-factor solution that corresponded to the three subscales of the DASS-21. The Heterotrait-Monotrait (HTMT) correlation ratio was then used to assess the discriminant validity, with good results. Results showed that the DASS21 has satisfactory reliability indexes (Cronbach’s α > 0.90).

**Conclusions:**

In light of the notable changes in living conditions brought by the COVID-19 pandemic, the present study has shown that the DASS-21 instrument has maintained its reliability and validity. Consequently, this finding supports the appropriateness of using the DASS-21 as a screening tool for assessing mental health among students in Portugal. Moreover, it is recommended that academics and healthcare practitioners use the DASS-21 as a tool for assessing the levels of psychological distress experienced by students. Additional validation studies of this scale are required, using bigger and more representative populations.

**Disclosure of Interest:**

None Declared

